# CMHX008, a Novel Peroxisome Proliferator-Activated Receptor γ Partial Agonist, Enhances Insulin Sensitivity *In Vitro* and *In Vivo*

**DOI:** 10.1371/journal.pone.0102102

**Published:** 2014-07-08

**Authors:** Yue Ming, Xiangnan Hu, Ying Song, Zhiguo Liu, Jibin Li, Rufei Gao, Yuyao Zhang, Hu Mei, Tingwang Guo, Ling Xiao, Bochu Wang, Chaodong Wu, Xiaoqiu Xiao

**Affiliations:** 1 Laboratory of Lipid & Glucose Metabolism, The First Affiliated Hospital of Chongqing Medical University, Chongqing, China; 2 College of Pharmacy, Chongqing Medical University, Chongqing, China; 3 Department of Endocrine, The First Affiliated Hospital of Chongqing Medical University, Chongqing, China; 4 Department of Nutrition and Food Hygiene, School of Public Health and Management, Chongqing Medical University, Chongqing, China; 5 College of Bioengineering, Chongqing University, Chongqing, China; 6 Department of Nutrition and Food Science, Texas A&M University, College Station, Texas, United States of America; Philipps University, Germany

## Abstract

The peroxisome proliferator-activated receptor γ (PPARγ) plays an important role in adipocyte differentiation and insulin sensitivity. Its ligand rosiglitazone has anti-diabetic effect but is frequently accompanied with some severe unwanted effects. The aim of the current study was to compare the anti-diabetic effect of CMHX008, a novel thiazolidinedione-derivative, with rosiglitazone. A luciferase assay was used to evaluate *in vitro* PPARγ activation. 3T3-L1 cells were used to examine adipocyte differentiation. High fat diet (HFD) mice were used to examine *in vivo* insulin sensitivity. The mRNA levels were evaluated by real-time RT-PCR. Serum biochemical and hormonal variables were assessed using a clinical chemistry analyser. CMHX008 displayed a moderate PPARγ agonist activity, and promoted 3T3-L1 preadipocyte differentiation with lower activity than rosiglitazone. CMHX008 regulated the expression of PPARγ target genes in a different manner from rosiglitazone. CMHX008 increased the expression and secretion of adiponectin with the similar efficacy as rosiglitazone, but only 25% as potent as rosiglitazone for the induction of adipocyte fatty acid binding protein. Treatment of CMHX008 and rosiglitazone protected mice from high fat diet (HFD)-induced glucose intolerance, hyperinsulinemia and inflammation. CMHX008 reduced the mRNA expression of M1 macrophage markers, and significantly increased the expressions of M2 markers. In conclusion, CMHX008 shared the comparable insulin-sensitizing effects as rosiglitazone with lower adipogenic capacity and might potentially be developed into an effective agent for the treatment of diabetes and metabolic disorders.

## Introduction

Peroxisome proliferator-activated receptor γ (PPARγ) is a ligand-activated transcription factor that belongs to a subfamily of nuclear hormone receptors and plays a pivotal role in the regulation of glucose and lipid homeostasis [1]–[3]. It is predominantly expressed in adipose tissue and plays a central role in adipose tissue functions. PPARγ is the master regulator of adipocyte differentiation, and enhances the numbers of insulin-sensitive small adipocytes [4], [5]. Its ligands, thiazolidinediones (TZDs), such as rosiglitazone and pioglitazone, are highly effective in treating insulin resistance and type 2 diabetes mellitus (T2DM) [6]–[8]. Unfortunately, TZDs are associated with serious safety issues, including weight gain, edema, increased incidence of heart attack, and bone loss [9]–[12]. Even though Food and Drug Administration has recently removed some prescribing and dispensing restrictions for rosiglitazone-containing diabetes medicines, the concern on the safety of TZDs remains [13]. As PPARγ agonism is the only available efficacious method to improve peripheral insulin sensitivity, medications of this class remain highly interesting for treatment of T2DM, which underscores the need for new leading compounds that achieve insulin sensitization without severe undesirable adverse effects.

Previously, Yamauchi et al reported that optimal approach to achieve insulin sensitization did not need full agonism of PPARγ [14], [15]. In supporting this notion, human carriers of a Pro12Ala polymorphism in the PPARγ gene, which causes reduced PPARγ activity, displayed improved insulin sensitivity [16]. In addition, PPARγ heterozygous null mice exhibited improved insulin sensitivity [17]. On the other hand, evidence has showed that powerful activation of PPARγ leads to adipocyte differentiation and increased adipose tissue mass, contributing to the weight gain. This is an important unwanted effect of TZDs. Increasing doses of TZDs produced both greater benefits for glucose control and greater incidence and higher degrees of side effects [18]. Therefore, explorations of novel PPARγ ligands with moderate modes of activation are required for the development of therapeutic agents for insulin resistance and T2DM.

**Figure 1:**
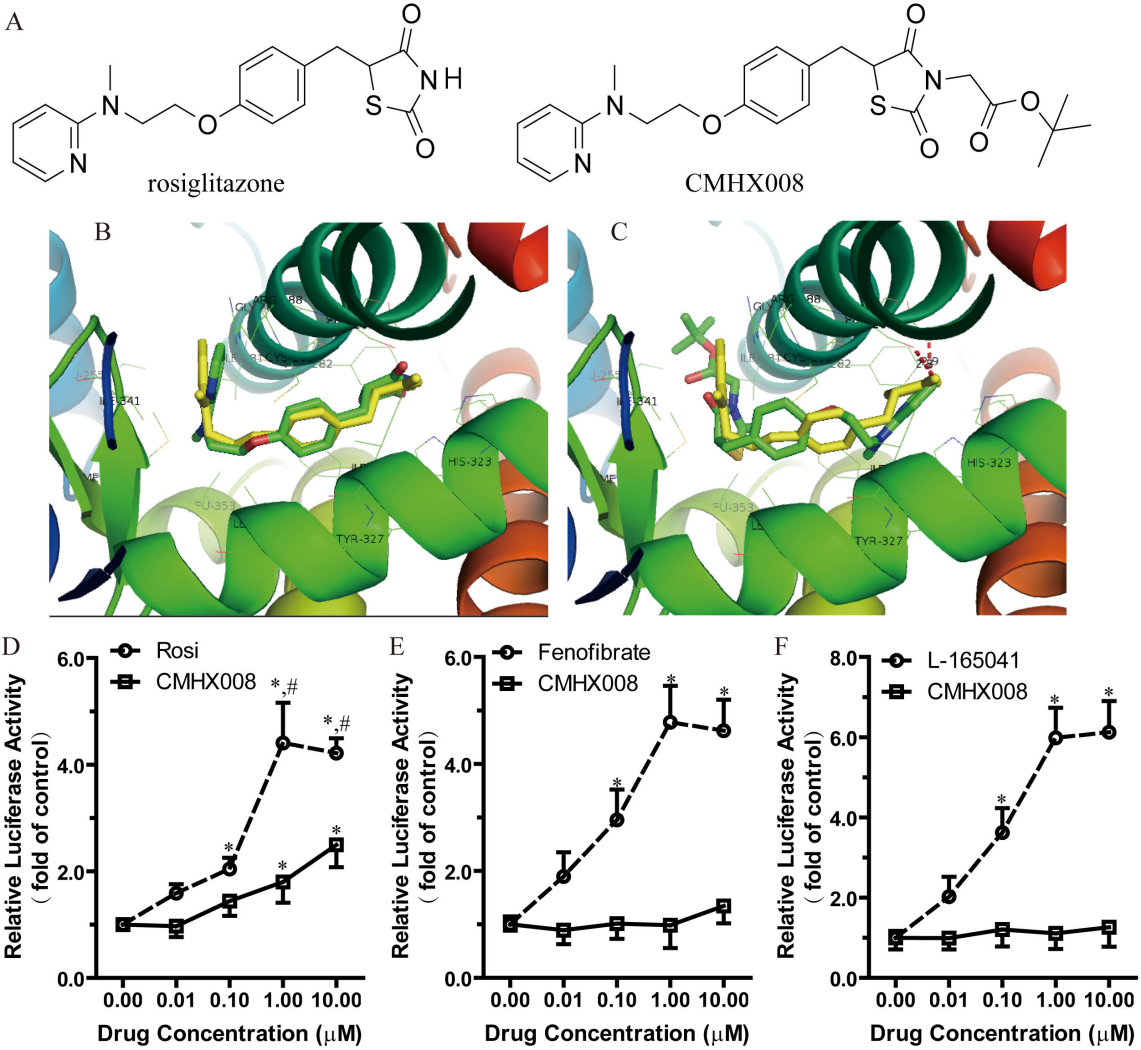
CMHX008 interacts and activates PPARγ activity in a different manner from rosiglitazone. A. Chemical structure of rosiglitazone and CMHX008. B. The optimal docking conformer of rosiglitazone (Yellow: crystal structure; Green: docking conformer). C. The optimal docking conformer of CMHX008 (Yellow: crystal structure of rosiglitazone; Green: docking conformer of CMHX008). Effects of CMHX008 on PPARγ (D), PPARα (E), and PPARβ/δ (F) activities in luciferase reporter assays. HEK293 cells were transfected with pIRES-hPPARs-EGFP, pPPRE×3-TK-Luciferase and pRL-TK. Cells were incubated in a medium with CMHX008 and rosiglitazone (D), or fenofibrate (E) or L-165041 (F) or 0.05% of DMSO (vehicle) for another 24 h after the transfection. The activity of vehicle was set as 1 and the obtained relative luciferase activities were presented as the fold induction with respect to that in the vehicle. All of the values are the means ± S.E.M. from 4–6 independent experiments. *P<0.05 *vs* vehicle, and ^#^ *P<*0.05 *vs* Rosi.

Based on the efficacy that ligands exhibit in cell based transactivation assays, PPARγ agonists are classified as either full or partial agonists depending upon the maximal efficacy on activating PPARγ [19]. TZDs were represented as full agonists and those PPARγ agonists with low activity than full agonists at saturating concentrations were represented as partial agonists. Compared with TZDs, several partial PPARγ agonists exhibited improved safety margins and consequently more efforts have been put into these promising partial PPARγ agonists for clinical development [20]–[23]. Using luciferase reporter assay, we identified a novel thiazolidinedione-derivative, CMHX008 (Hu X, Xiao X, Liu Z, *et al*, Preparation and biological functions of novel thiazolidinedione-derivatives, Chinese Patent ID 201210202455.2), chemically known as 3-tert-butoxycarbonylmethyl-5-[4-[2-[N-methyl-N-(2-pyridyl)amino]-ethoxy]benzyl] thiazolidine-2, 4-dione (Figure 1A), as a PPARγ partial agonist, with lower activity of PPARγ agonism than rosiglitazone. CMHX008 promotes 3T3-L1 cell differentiation in a PPARγ dependent manner, and possibly shares the similar insulin-sensitizing effects as rosiglitazone but with lower adipogenic capacity. Accordingly, CMHX008 protected mice from high fat diet-induced insulin resistance and repressed transcription of genes involved in chronic inflammation. These data indicate that CMHX008 may potentially be developed into an effective and relatively-safe molecule for the treatment of T2DM and metabolic disorders.

## Materials and Methods

### Materials

3T3-L1 preadipocytes were obtained from ATCC. Dulbecco’s modified Eagle’s medium (DMEM), penicillin-streptomycin and fetal bovine serum (FBS) were purchased from HyClone. Bovine calf serum was from Gibco. Paraformaldehyde, Oil Red O, 3-isobutylmethylxanthine (IBMX), dexamethasone (DEX), insulin, GW9662, fenofibrate and L-165041 were purchased from Sigma-Aldrich (St Louis, MO). Lipofectamine 2000 was obtained from Invitrogen. SYBR Green was from BIO-RAD. Rosiglitazone was a kind of gift from Chongqing Taiji Industry (Group) Co. Ltd. The synthesis of CMHX008 has been described in our recent patent in China. C57BL/6 mice were obtained from the Jackson Laboratory (Vital River, Beijing), and rodent low fat diet (LFD, Cat.# D12450B, 19.2% protein, 4.3% fat, and 67.3% carbohydrate, and 10% of calories from fat) or high-fat diet (HFD, Cat.# D12451, 24% protein, 24% fat, and 41% carbohydrate, and 45% calories from fat) were purchased from Shanghai SLAC Laboratory Animal CO. LTD. (Shanghai, China). All other reagents were of analytical purity grade.

### Molecular docking study of CMHX008 with human PPARγ (hPPARγ)

To determine the binding model of PPARγ and CMHX008, Surflex-dock was performed by using Sybyl 8.1 package. Surflex-dock is a fully automatic flexible molecular docking algorithm based on molecular similarity and an empirical-based scoring function [24]. The docking score is expressed in -lg (Kd) unit, which consists of hydrophobic, polar, electrostatic, repulsive, entropic, and solvation terms. The crystal structure of PPARγ was derived from PDB database (ID: 2PRG). Herein, the co-crystal ligand, rosiglitazone was used to generate a protocol with the threshold and bloat of 0.30 and 2.0 Å, respectively. Prior to docking, CMHX008 were optimized by Tripos force field and MMFF94 partial charges. The number of additional starting conformations and resulting docking poses were set to 5 and 20, respectively.

### Luciferase reporter gene assay

For luciferase assays, pPPRE×3-TK-Luciferase reporter plasmid, pRL-TK (an internal control plasmid for normalizing transfection efficiencies) and pIRES-hPPARγ-EGFP, or pIRES-hPPARα-EGFP, or pIRES-hPPARβ/δ-EGFP (human PPARγ, PPARα and PPARβ/δ expression vectors), were transfected into HEK293 cells by using Lipofectamine 2000 (Invitrogen) according to the manufacturer’s protocol. After 5 h of transfection, the cells were treated with rosiglitazone or CMHX008. After 24 h, cells were harvested and a luciferase assay was performed using the dual-luciferase system (Promega) according to the manufacturer’s instruction. Relative luciferase activity was normalized by the corresponding Renilla luciferase activity.

### 3T3-L1 cell culture and adipocyte differentiation and Oil Red O staining

3T3-L1 preadipocytes were cultured at 37°C in a humidified atmosphere of 5% CO2. Differentiation into adipocytes was performed according to standard protocols [25]. Briefly, the cells were maintained in growth medium containing DMEM with 10% bovine calf serum and 1% penicillin-streptomycin. For 3T3-L1 preadipocyte differentiation, cells after reaching confluency (defined as day 0) were cultured in differentiation medium comprising DMEM supplemented with 0.5 mM IBMX, 1 µg/ml insulin, 0.25 µM DEX and 10% FBS with indicated compounds. After 2 days, the culture medium was changed to DMEM containing 1 µg/ml insulin and 10% FBS. The medium was replaced again with fresh DMEM containing 10% FBS after another 3 days. Rosiglitazone, CMHX008 and GW9662 were dissolved in DMSO and the final DMSO concentrations were less than 0.1% in the medium. Rosiglitazone or CMHX008, with or without GW9662, was administered at the initiation of differentiation and added with every medium change. At day 7, the differentiated cells or cell media were harvested for the following experiments. After differentiation, cells were fixed with 4% paraformaldehyde for 30 min, rinsed with PBS twice, and stained with 0.5% Oil Red O working dye for 1 h. After washing again with PBS, the cells were photographed under a microscope (100×magnification). After staining, Oil Red O was extracted using isopropanol, and the absorbance was measured at a wavelength of 490 nm using a spectrophotometer [26].

### Animals and drug treatments

All animal studies were performed in accordance with the Guide for the Care and Use of Laboratory Animals of the National Institutes of Health. All experimental procedures were approved by the Institutional Animal Care and Use Committee of Chongqing Medical University. Male C57BL/6 mice (6-week-old) were housed under standard conditions with a 12 h/12 h light-dark cycle and constant temperature (23±2°C). Mice were fed either HFD or a control LFD. After 16 weeks for HFD, mice were administered once daily with CMHX008 (3 or 10 mg/kg/day), rosiglitazone (3 mg/kg/day) or an equivalent volume of vehicle by intragastric gavage for additional 5 weeks. The drugs were suspended in 0.5% methylcellulose, and during the drug treatment, mice were maintained at the same HFD or LFD diets. At the end of the study, all mice were euthanatized by CO2 inhalation and killed in a non-fasting state. Trunk blood and epididymal white adipose tissues (WAT) were collected.

### Intra-peritoneal glucose tolerance tests (iGTTs)

Four weeks after drug treatment, iGTTs were performed for portions of the animals. Mice were fasted overnight, and then 20% glucose solution was injected intra-peritoneally at 2 g/kg. The time-point before glucose injection was set as 0 min, and blood glucose concentrations at 0, 15, 30, 60 and 120 min were measured using a glucometer (SureStep OneTouch, USA).

### RNA preparation and quantitative real-time PCR

Total RNA was isolated from differentiated cells or WAT using Trizol reagent (Invitrogen) and 1 µg of total RNA from each sample was reverse-transcribed to cDNA using random primers and a reverse transcription system (Takara) according to the manufacturer’s protocol. After cDNA synthesis, quantitative real-time PCR was performed using iQ SYBR Green Supermix (Bio-Rad) according to the manufacturer’s instructions. The PCR conditions were 1 cycle of 95°C for 3 min, followed by 39 cycles of 95°C for 10s and 60°C for 30s. PCR reactions were carried out in a 25 µl reaction buffer that included 12.5 µl SYBR Green, 0.25 µl of forward primer, 0.25 µl of reverse primer, 2 µl of cDNA, and 10 µl ddH2O and performed in triplicate for each sample in the BIO-RAD CFX96 Real-Time PCR system. The fluorescence intensity of each sample was measured at each temperature change to monitor amplification of the target gene. The comparative cycle time (CT) method was used to determine fold differences between samples. The comparative CT method determined the amount of target normalized to an endogenous reference (GAPDH) and relative to a calibrator. The primer sequences used for the PCR amplification were as follows: GAPDH, 5′-CAA GGT CAT CCA TGA CAA CTT TG-3′ and 5′-GGC CAT CCA CAG TCT TCT GG-3′; adiponectin, 5′-GAC ACC AAA AGG GCT CAG GAT-3′ and 5′-TGG GCA GGA TTA AGA GGA ACA-3; aP2, 5′-CAA CCT GTG TGA TGC CTT TGT G-3′ and 5′-CTC TTC CTT TGG CTC ATG CC-3′; Glut4, 5′-CAT GGC TGT CGC TGG TTT C-3′ and 5′- AAA CCC ATG CCG ACA ATG A-3′; CD11c, 5′-CGGCTGGATCAGTCAGACATT-3′ and 5′-CGATAAGAGGGACGGGGTT-3′; CD206, 5′-ACAAAGGGACGTTTCGGTG-3′ and 5′-TGGACATTTGGGTTCAGGAG-3′; MCP-1, 5′-TGTGCTGACCCCAAGAAGG-3′ and 5′-TGAGGTGGTTGTGGAAAAGG-3′; arginase-1, 5′-AACACTCCCCTGACAACCA-3′ and 5′-CATCACCTTGCCAATCCC-3′; inducible nitric oxidase (iNOS), 5′-CACGGACGAGACGGATAG-3′ and 5′-CACTGACACTTCGCACAAA-3′.

### Histological analysis

For haematoxylin-eosin (H&E) staining, epididymal fat tissues were dissected and fixed in 4% paraformaldehyde for 72 h, dehydrated using gradient ethanol and embedded in paraffin. Tissue sections were cut at 5 µm in thickness and stained with H&E. The tissue slides were examined using Nikon Optical Microscope and pictures were taken and analyzed using Nikon NIS-Elements BR software. Analysis of adipocites was based on the previous report [27]. Sizes of adipocytes from 3 representative tissue areas per mouse were calculated to obtain the average adipocyte size and represented as the fold change of LFD-V.

### Measurement of cytokines, leptin and insulin

Adiponectin levels in the culture medium of differentiated 3T3 L1 adipocytes and serum were measured using mouse adiponectin ELISA kit (CUSABIO, Wuhan, China). Others mouse cytokines, leptin and insulin were detected by Milliplex Map Multiplex Assay (Merck Millipore, Billerica, MA, USA) according to the manufacturer’s protocol.

### Serum biochemical assays

Serum triglyceride (TG), total cholesterol (TC), high-density lipoprotein (HDL), low-density lipoprotein (LDL), and aspartate aminotransferase (AST) and alanine aminotransferase (ALT) were measured with Biochemical Analyzer (SIEMENS ADVIA 2400 Chemisty Systerm, Germany).

### Statistics analysis

Data are expressed as the mean ± S.E.M. One-way ANOVA, followed by Newman-Keuls's multiple range test, was used to determine significant differences among groups. Two group comparison was performed using unpaired Student *t*-test. Statistical analyses were conducted by using GraphPad Prism software (GraphPad Prism, San Diego, CA, USA), and statistical significance was defined as *P*<0.05.

## Results

### CMHX008 interacts and activates PPARγ activity in a different manner from rosiglitazone

To validate the Surflex-dock method employed in the current study, rosiglitazone was firstly docked into the binding site of PPARγ receptor by using the same docking protocol as CMHX008. Figure 1B showed that an optimal docking conformer of rosiglitazone (Total score = 8.69) was aligned with the crystal structure (RMSD = 0.25Å). Figure 1C showed an optimal docking conformer (Total score = 8.52) of CMHX008. It is apparent that the binding mode of CMHX008 (green) was quite different from that of rosiglitazone (yellow). In the case of rosiglitazone, the thiazolidinedione ring was located in the bottom of the binding pocket with H-bond interactions with Gln286 and Ser289, while the pyridine group in the entrance. However, in the case of CMHX008, the pyridine group was inverted to the bottom of the pocket leaving the thiazolidine ring in the entrance. Also, the CH2COOC(CH3)3 group formed hydrophobic interactions with Leu255, Ile281, Met348, and Ile341 in the entrance.

To investigate whether CMHX008 enhances PPARγ activity, we performed a luciferase reporter assay using full-length PPARγ and PPRE×3-TK-Luciferase expression vectors. PPARγ luciferase activity was enhanced by rosiglitazone treatment in a dose dependent manner with the highest efficacy at 1.0 µM and enhanced by 3.4-folds compared with vehicle (Figure 1D). However, in the presence of CMHX008, PPARγ activity was increased by 0.8 and 1.5-folds at the concentrations of 1.0 and 10 µM, respectively. The maximal PPARγ luciferase activity induced by CMHX008 was approximately 45% of that induced by rosiglitazone, indicating that CMHX008 was a moderate PPARγ ligand (Figure 1D). Compared with fenofibrate and L-165041, which were specific agonists of PPARα and PPARβ/δ respectively, CMHX008 did not alter PPARα and PPARβ/δ activities (Figure 1E and 1F).

### CMHX008 enhances adipocyte differentiation in 3T3-L1 cells through activating PPARγ

**Figure 2:**
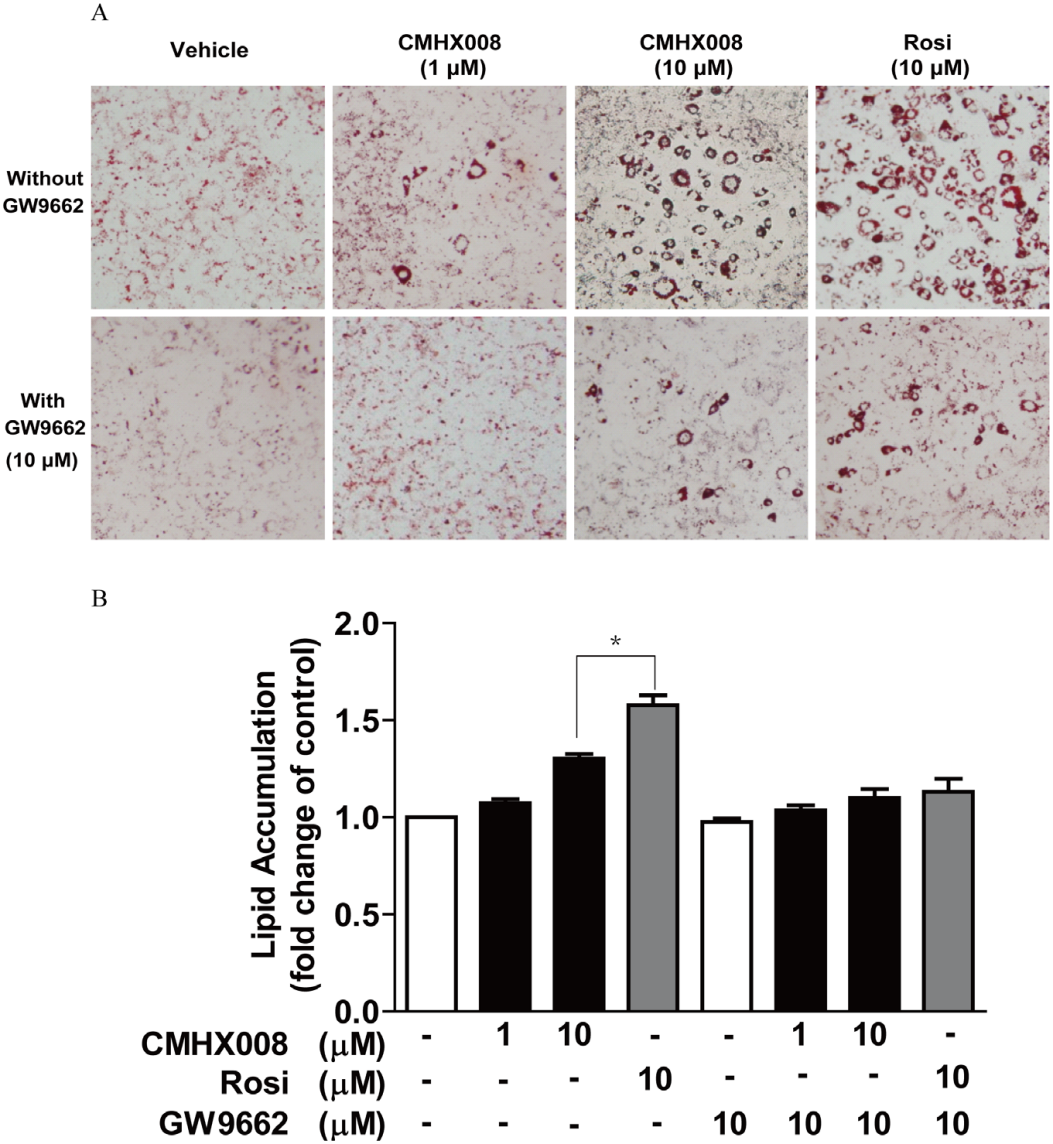
CMHX008 promotes 3T3-L1 preadipocytes differentiation and lipid accumulation. Post-confluent 3T3-L1 preadipocytes were incubated in differentiation medium for 2 days. The medium was changed to DMEM containing insulin and 10% FBS for another two days and then replaced again with fresh DMEM containing 10% FBS for another 3 days. Rosiglitazone and CMHX008, with or without GW9662, were administered at the initiation of differentiation and added with every medium change. At day 7, the differentiation cells were used for Oil Red O staining. A. The cells were fixed and stained with Oil Red O, and then stained adipocytes were photographed (100×); B. Stained cells were eluted with isopropyl alcohol and quantified at 490 nm. Data are the means ± S.E.M. from five experiments. *: *P<*0.05.

As shown in Figure 2, CMHX008 at 10 µM significantly promoted adipocyte differentiation and increased intracellular lipid accumulation. However, compared to rosiglitazone, CMHX008 was less effective on promoting adipocyte differentiation and increasing intracellular lipid accumulation (Figure 2A and 2B). These results suggest that CMHX008 promotes differentiation of 3T3-L1 preadipocytes, but the intensity is weaker than rosiglitazone.

To confirm CMHX008 as a PPARγ ligand, we examined the effect of GW9662, a PPARγ specific antagonist, on CMHX008-induced 3T3-L1 preadipocyte differentiation. Co-treatment with GW9662 resulted in a significant inhibition on 3T3-L1 preadipocyte differentiation (Figure 2A) and lipid accumulation (Figure 2B) induced by rosiglitazone and CMHX008. Taken together, these findings suggest that CMHX008 enhances adipocyte differentiation and lipid accumulation in a PPARγ dependent manner.

### CMHX008 differentially regulates PPARγ target genes

**Figure 3:**
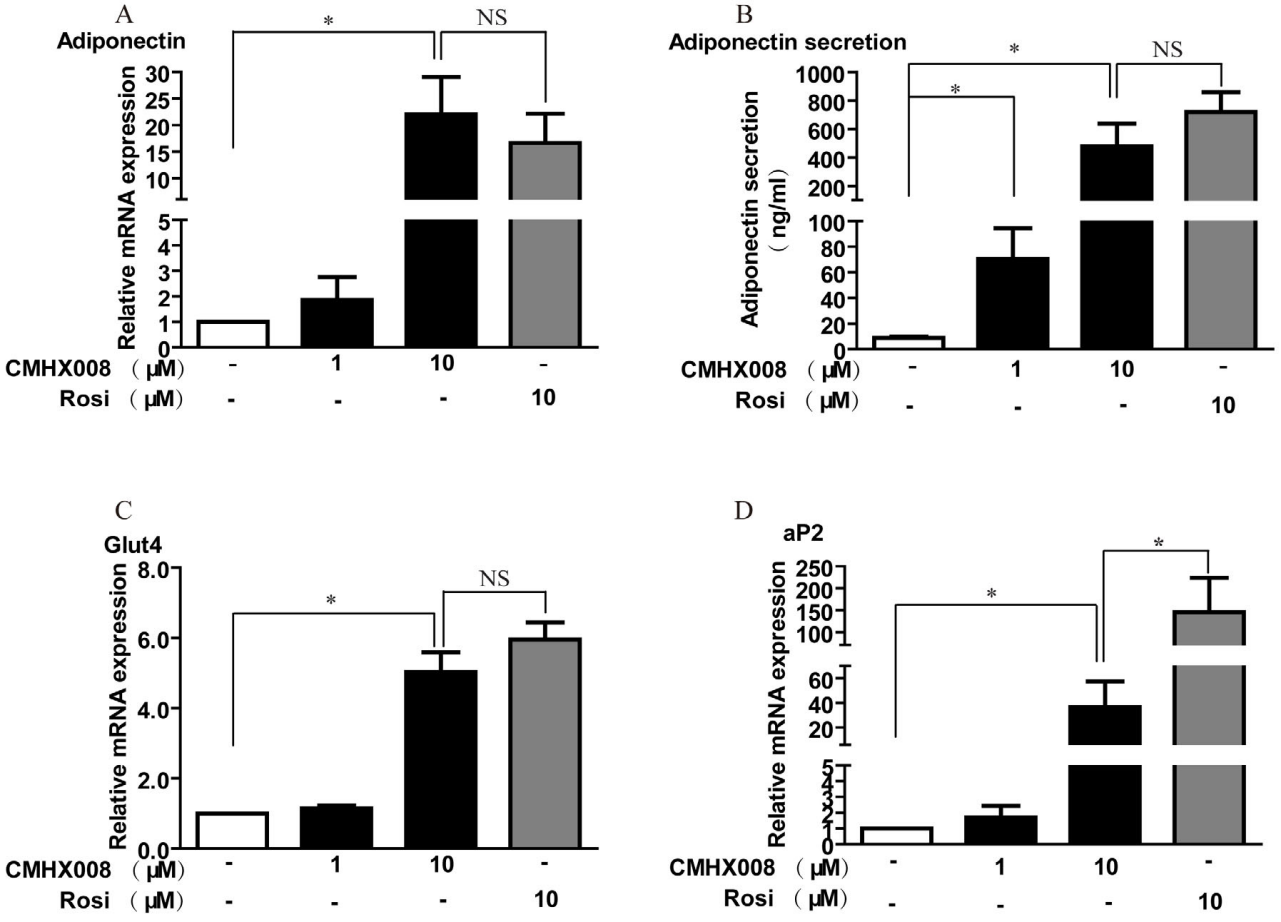
CMHX008 differentially regulates PPARγ target genes in 3T3-L1 adipocytes. Post-confluent 3T3-L1 preadipocytes were incubated in differentiation medium for 2 days. The medium was changed to DMEM containing insulin and 10% FBS for another two days and then replaced again with fresh DMEM containing 10% FBS for another 3 days. Rosiglitazone and CMHX008 were administered at the initiation of differentiation and with every medium change. At day 7, the differentiation cells were harvested for total RNA extraction and the mRNA expression of adiponectin (A), Glut4 (B) and aP2 (D) was analyzed by real-time PCR, and secreted adiponectin in the culture medium was measured using adiponectin ELISA kit (B). Data are the means ± S.E.M. from six independent experiments. *: *P<*0.05; NS: no significance.

To elucidate whether CMHX008 induces expression of PPARγ target genes during the adipocyte differentiation, we performed quantitative real-time PCR analysis of well characterized PPARγ target genes adiponectin, fatty acid binding protein 4 (aP2) and glucose transporter 4 (Glut4). As shown in Figure 3A, the expression of adiponectin after treatment with CMHX008 was similar to rosiglitazone, suggesting that it might have similar insulin sensitizing effect as rosiglitazone. CMHX008 increased the expression of Glut4, and the potency was close to rosiglitazone (Figure 3C). Both CMHX008 and rosiglitazone can significantly upregulate the mRNA expression of aP2, but CMHX008 showed only 25% as potent as rosiglitazone for the induction of aP2 (Figure 3D). Meanwhile, the measurement of adiponectin secretion (Figure 3B) was in agreement with mRNA levels of adiponectin. These results indicated that CMHX008 appears to have differential selectivity towards PPARγ target genes.

### CMHX008 protected diet induced metabolic abnormalities in vivo

**Figure 4:**
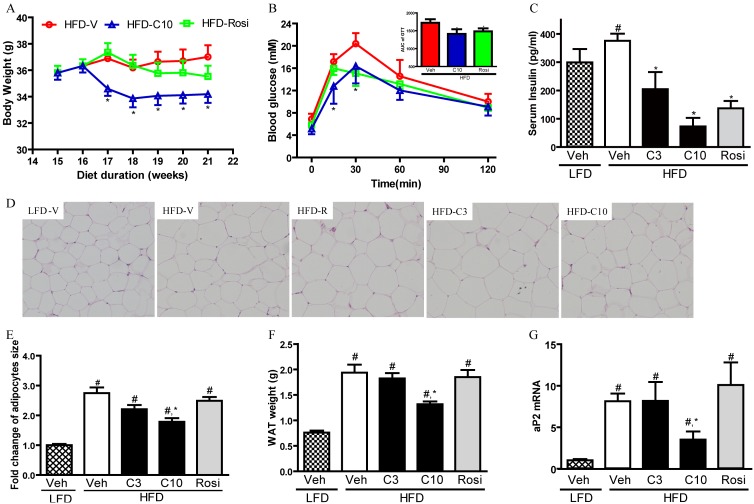
CMHX008 protects HFD induced obesity and insulin resistance. C57BL/6 mice were fed with HFD or LFD for 16 weeks. Then HFD fed C57BL/6 mice were treated with CMHX008 (3 and 10 mg/kg, HFD-C3 and HFD-C10), rosiglitazone (3 mg/kg, HFD-Rosi) or vehicle (HFD-Veh or LFD-Veh) only. After 4 weeks treatment, intra-peritoneal glucose tolerance tests (iGTTs) were performed (B) and area under curve (AUC) of iGTTs was calculated (B, inserted Figure). Changes in body weight (A), fasting insulin levels (C), morphology of adipocytes (D), quantified adipocyte size (E), mass of epididymal adipose tissues (WAT, F) and aP2 mRNA levels (G) were examined. Data are the means ± S.E.M., n = 6–8; ^#^ *P<*0.05 *vs* LFD-Veh and *P<0.05 *vs* HFD-Veh.

We then further evaluated the effect of CMHX008 and rosiglitazone on HFD-induced insulin resistance in C57BL/6 mice. At the dose of 3 mg/kg, CMHX008 (data not shown) and rosiglitazone did not change the body weight while 10 mg/kg of CMHX008 slightly reduced the body weight (Figure 4A). Fifteen and 30 min after glucose loading, plasma glucose levels and area under curve (AUC) of iGTTs in 10 mg/kg of CMHX008 treated mice were significantly lower than those in vehicle-treated mice. In rosiglitazone-treated mice, plasma glucose levels at 30 min after glucose loading and AUC were lower than vehicle treated mice (Figure 4B) CMHX008 at 3 mg/kg did not affect iGTTs compared with vehicle-treated mice (data not shown). In addition, both rosiglitazone and CMHX008 significantly improved HFD-induced hyperinsulinemia (Figure 4C). In accordance with changes in body weight, 10 mg/kg of CMHX008 significantly reduced the size of adipocytes, decreased the WAT weight and downregulated aP2 mRNA expression in WAT (Figure 4D–4G), suggesting CMHX008 prevented HFD-induced hypertrophy in adipocytes. These results indicated that CMHX008 at 10 mg/kg might have a similar anti-diabetic effect as rosiglitazone with lower adipogenic capacity.

**Figure 5:**
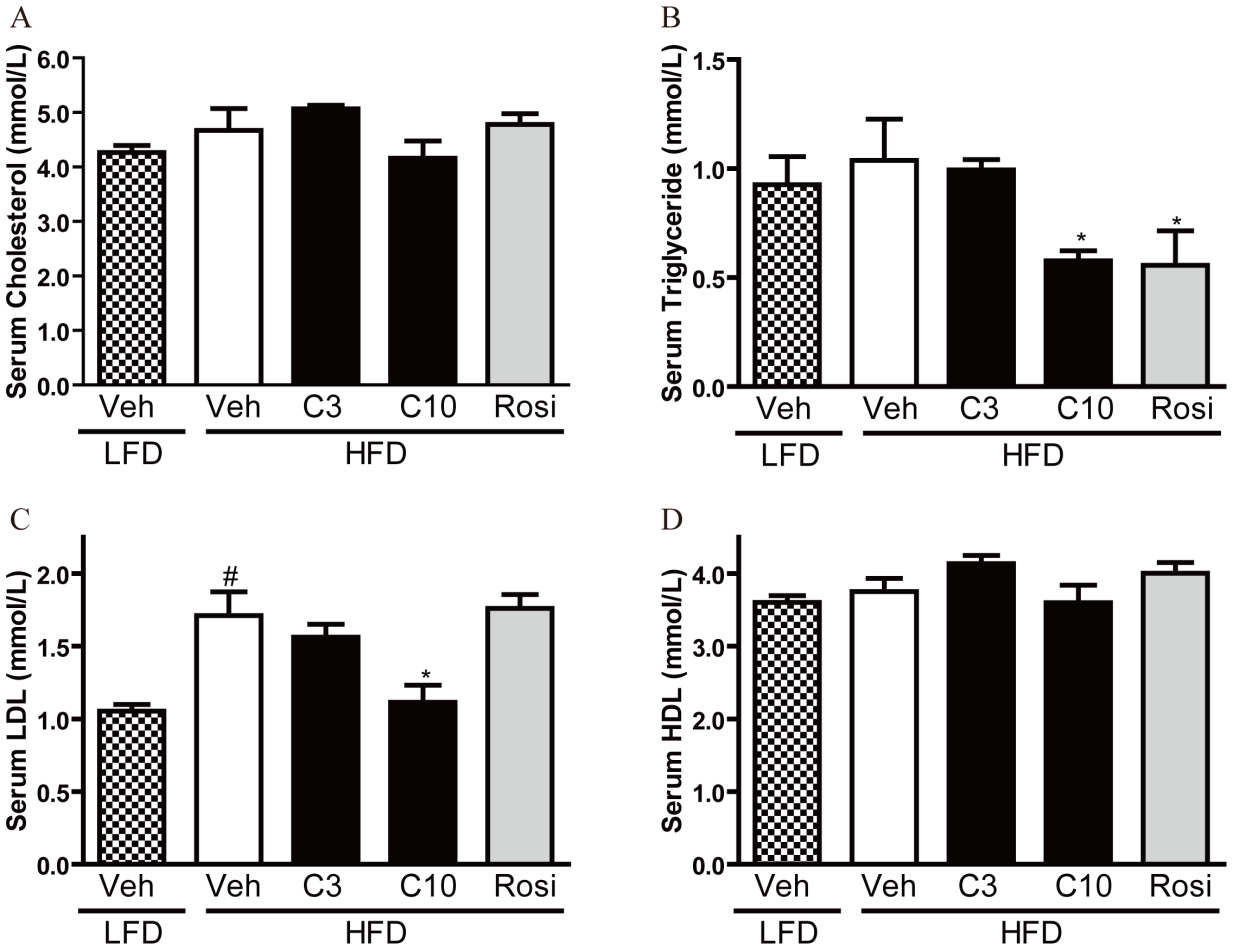
CMHX008 protects HFD induced dyslipidaemia. C57BL/6 mice were fed with HFD or LFD for 16 weeks. Then HFD fed C57BL/6 mice were treated with CMHX008 (3 and 10 mg/kg, HFD-C3 and HFD-C10), rosiglitazone (3 mg/kg, HFD-Rosi) or vehicle (HFD-Veh or LFD-Veh) only, for additional 5 weeks. Trunk blood was collected at the end of the experiment. Serum was used for the measurement of cholesterol (A), triglyceride (B), low-density lipoproteins (LDL, C) and high-density lipoproteins (HDL, D). Data are the means ± S.E.M., n = 4–5; ^#^ *P<*0.05 *vs* LFD-Veh and *P<0.05 *vs* HFD-Veh.

We then investigated the effect of CMHX008 and rosiglitazone on HFD-induced changes in hormonal and biochemical characteristics in C57BL/6 mice. CMHX008 (10 mg/kg) significantly decreased serum levels of triglyceride (Figure 5B) and low-density lipoproteins (LDL) (Figure 5C), but did not affect levels of cholesterol (Figure 5A) and high-density lipoproteins (HDL) (Figure 5D).

The improvement of metabolic abnormalities by CMHX008 was associated with its anti-inflammatory effects.

**Figure 6:**
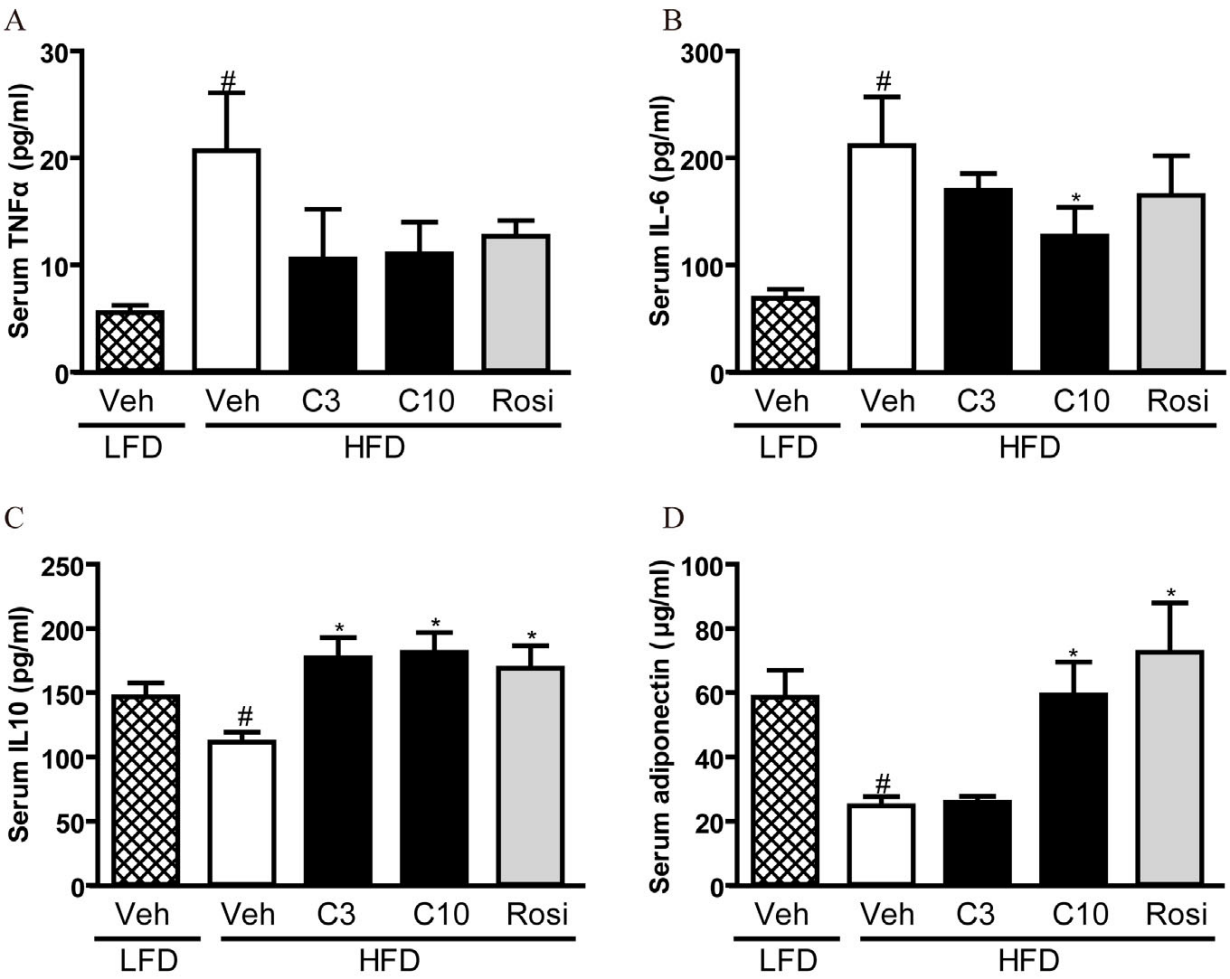
CMHX008 improves HFD induced systemic inflammation. C57BL/6 mice were fed with HFD or LFD for 16 weeks. Then HFD fed C57BL/6 mice were treated with CMHX008 (3 and 10 mg/kg, HFD-C3 and HFD-C10), rosiglitazone (3 mg/kg, HFD-Rosi) or vehicle (HFD-Veh or LFD-Veh) only, for additional 5 weeks. Trunk blood was collected at the end of the experiment. Serum was used for the measurement of TNF-α (A, IL-6 (B), IL-10 (C), and adiponectin (D). Data are the means ± S.E.M., n = 4–5; ^#^ *P<*0.05 *vs* LFD-Veh and *P<0.05 *vs* HFD-Veh.

To further understand the underlying mechanism, we measured serum levels of inflammatory and anti-inflammatory cytokines. As shown in Figure 6A and 6B, TNF-α and IL-6, two critical inflammatory mediators, were significantly increased in HFD fed mice and 10 mg/kg of CMHX008 reduced IL-6 levels. In contrast, interleukin-10 (IL-10), a cytokine with broad anti-inflammatory properties, was lowered in HFD mice and treatment with CMHX008 and rosiglitazone significantly increased IL-10 levels (Figure 6C). The similar change pattern was observed for serum adiponectin levels (Figure 6D). These findings indicated that the antidiabetic activity of CMHX008 and rosiglitazone may be associated with their anti-inflammatory effects.

### The anti-inflammatory effects of CMHX008 was dependent on the change in macrophage polarization

**Figure 7:**
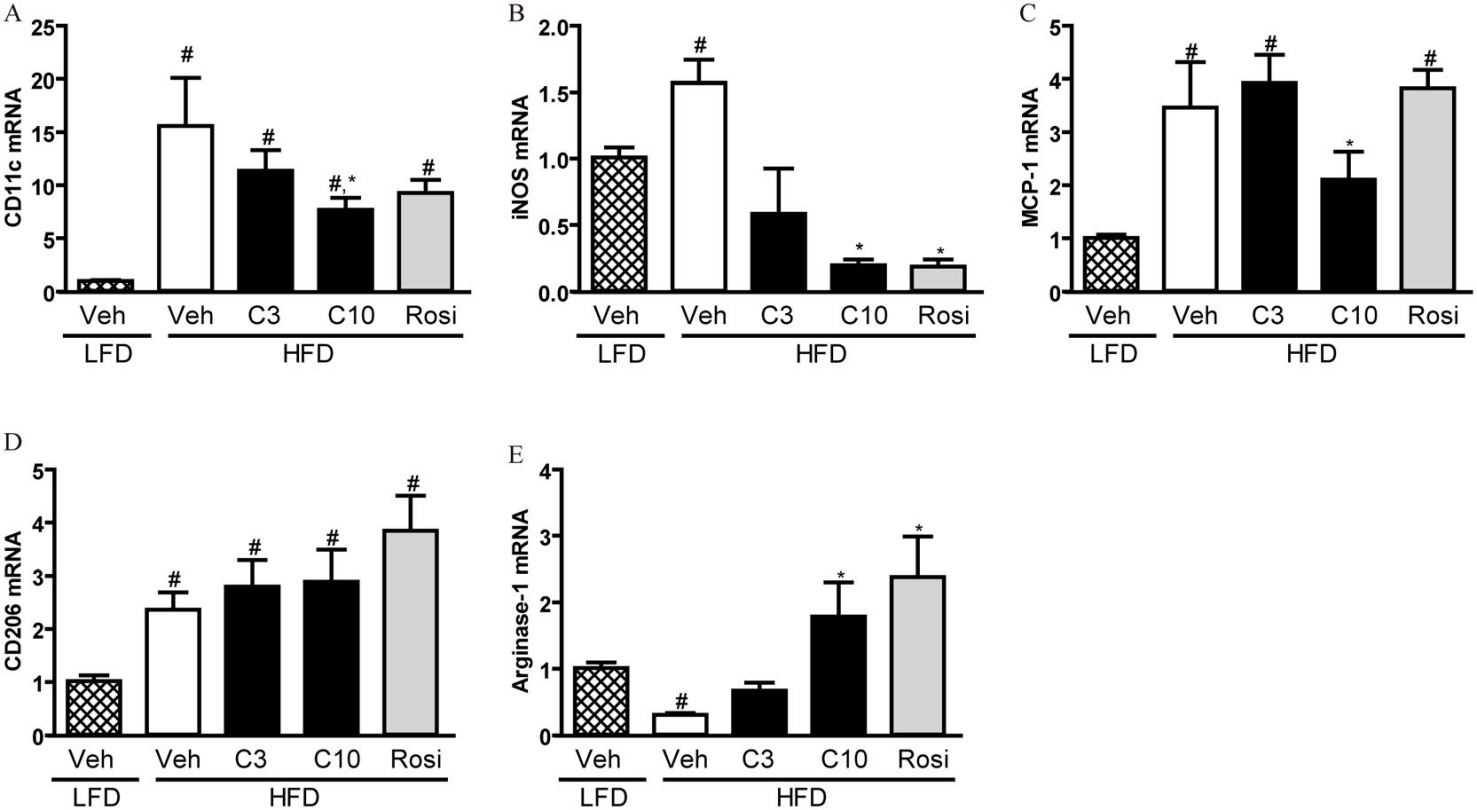
Anti-inflammatory effect of CMHX008 is dependent on the change in adipose tissue macrophage polarization. C57BL/6 mice were fed with HFD or LFD for 16 weeks. Then HFD fed C57BL/6 mice were treated with CMHX008 (3 and 10 mg/kg, HFD-C3 and HFD-C10), rosiglitazone (3 mg/kg, HFD-Rosi) or vehicle (HFD-Veh or LFD-Veh) only, for additional 5 weeks. Epididymal adipose tissues were harvested for total RNA extraction and the mRNA expression of CD11c (A), iNOS (B), MCP-1 (C), CD206 (D) and arginase-1 (E) was analyzed by real-time PCR. Data are the means ± S.E.M., n = 6–8; ^#^ *P<*0.05 *vs* LFD-Veh and *P<0.05 *vs* HFD-Veh.

We then examined whether the anti-inflammatory effects of CMHX008 was associated with the change of macrophage polarization. As shown in Figure 7A–7C, the expression of M1 macrophage markers CD11c, iNOS and MCP-1 was dramatically elevated by HFD-feeding, and the upregulation of CD11c, iNOS and MCP-1 was significantly attenuated by treatment with CMHX008 while rosiglitazone treatment decreased only iNOS upregulation. CD206 and arginase-1 were important markers of M2 macrophages. Interestingly, the mRNA levels of CD206 also increased by HFD-feeding compared with LFD-fed mice, and both CMHX008 and rosiglitazone further increased CD206 mRNA (Figure 7D). HFD-feeding dramatically repressed the expression of arginase-1, which was totally blocked by both CMHX008 and rosiglitazone (Figure 7E). These results indicate that administration of CMHX008 and rosiglitazone to HFD feeding mice switched the polarization of adipose macrophages from M1 dominant to M2 dominant.

## Discussion

TZDs are a class of PPARγ agonists used in the treatment of T2DM. However, TZDs are associated with numerous undesired side effects [9]–[11], [28], [29], which were generally considered due to excessive activation of PPARγ. Therefore, substantial efforts have been made to generate novel partial PPARγ agonists that retain the beneficial clinical effects while minimizing the unwanted side effects. With the aim to improve safety and maintain anti-diabetic efficacy of TZDs, we synthesized a new thiazolidinedione-derivative, CMHX008. In the current study, our *in vitro* data showed that CMHX008 enhanced PPARγ activity and promoted 3T3-L1 preadipocyte differentition with the intensity significantly lower than rosiglitazone. In addition, co-treatment with GW9662, a PPARγ specific antagonist, abolished the effect of CMHX008 on 3T3-L1 preadipocyte differentition, indicating that CMHX008 might bind to PPARγ and activate PPARγ as a ligand. Further analysis of PPARγ target genes exhibited beneficial effects of CMHX008 as it significantly upregulated insulin sensitization related genes (adiponectin and GluT4) as rosiglitazone. However, CMHX008 was only 25% as potent as rosiglitazone for the induction of aP2, an adipogenic gene which is a great predictive marker for the undesired side effects of TZDs associated with weight gain. In accordance, we observed a remarkable improvement on metabolic disturbance, along with beneficial effects on body weight change induced by CMHX008 in mice fed with HFD. Most notably, we found the protective effect of CMHX008 was due to its capacity to switch the polarization of adipose macrophages from M1 dominant to M2 dominant. These results suggest that CMHX008 may improve insulin resistance by modulating macrophage polarization. The pharmacological profiles suggested CMHX008 might possibly work as an antidiabetic agent that maintains insulin-sensitizing actions but is possibly dissociated from the deleterious effects on weight gain of classical TZDs.

Adiponectin is a secretory protein produced by mature adipocytes and circulating levels of adiponectin are generally negatively related to BMI and insulin resistance, with higher plasma adiponectin levels in lean subjects and lower adiponectin levels in obese individuals. A variety of experimental data suggest that adiponectin has anti-diabetic, anti-inflammatory and anti-atherogenic effects [30]. Adiponectin can be transcriptionally activated by PPARγ agonists as the promoter region of adiponectin has a binding site for PPARγ/retinoid X receptor heterodimer. Administration of PPARγ agonists significantly ameliorates insulin sensitivity in mice with high fat diets and patients with T2DM and the improvement of insulin resistance was consistent with the increase in the plasma adiponectin levels [31], [32], and the protective effects of adiponectin has mainly been attributed to its anti-inflammatory action [33]. These results suggest that the ameliorative effect of PPARγ agonists on insulin resistance was, at least in part, mediated through upregulation of adiponectin. In the current study, we found that CMHX008 enhanced the expression and secretion of adiponectin in a similar pattern as rosiglitazone in the mature adipocytes although it displayed only a 45% of PPARγ agonist activity as rosiglitazone judged by luciferase reporter assay using full-length PPARγ plasmid. Additionally, administration of CHMX008 (10 mg/kg) and rosiglitazone (3 mg/kg) into mice with HFD displayed similar extent of increasing serum adiponectin levels. These results were in agreement with previous report that insulin sensitization and antidiabetic activity did not depend upon the full agonism of PPARγ [14], [15], [34].

Excess agonism of PPARγ may possibly contribute to some types of adverse effects of TZDs [3], [35], [36]. aP2 plays an important role in adiposity associated with PPARγ activation, and therefore, the effect of PPARγ agonists on the induction of aP2 gene expression has been recognized as a key predictive parameter for their undiserable effects associated with adiposity [37]–[39]. In the present study, rosiglitazone induced a significant upregulation of aP2 expression, with strong adipogenic capacity and high degree of triglyceride accumulation in differentiated 3T3-L1 adipocytes. In contrast, induction of aP2 expression by CMHX008 was only 25% as rosiglitazone. Treatment with 10 mg/kg of CMHX008 decreased body weight in HFD feeding mice while rosiglitazone did not affect the body weight [40], [41]. These data suggest that CMHX008 could differentially regulate PPARγ target genes, and its optimal induction on adiponetin and GluT4 but low agonism on aP2 indicated that CMHX008 may have a similar effects of insulin sensitization as rosiglitazone *in vivo* and can improve adiposity, which was not observed for rosiglitazone.

The discrepancy of weight gain caused by rosiglitazone in clinical observation and experimental rodents had been reported by several studies [40]–[43]. At least, two main reasons may contribute to the changes in the food intake and body weight after treatment with PPARγ agonists. Firstly, the animals are not eating in a usual manner due to daily gavage, and the treatment per se may alter feeding behaviour and final body weight. Thus, supplement of these drugs in the daily food, instead of forced administration by gavage, may possibly reflect a physiological change in feeding and weight. Secondly, weight gain of rosiglitazone treatment may depend on the dose: at a higher dose (>10 mg/kg), rosiglitazone increased body weight, but no significant change in body weight while low dose (3 mg/kg) of this drug was given [42], which is consistent with our results.

One of the most important characteristics of obesity-induced metabolic disorders is low-grade chronic inflammation [44]. The recruitment of all types of inflammatory cells such as monocytes and natural killer T (NKT) cells, and differentiation and polarization to classically activated pro-inflammatory M1 macrophages instead of anti-inflammatory M2 macrophages, have been causally linked to the development of obesity and obesity-related metabolic abnormalities [45]–[48]. M1 macrophages produced and secreted high levels of proinflammatory cytokines such as monocyte chemoattractant protein-1 (MCP-1), tumor necrosis factor-α (TNF-α) and interleukin (IL)-6, and generated reactive oxygen species through the actions of inducible nitric oxide synthase (iNOS), with CD-11c antigen as its specific biomarker while M2 macrophage produced low levels of proinflammatory cytokines and high levels of anti-inflammatory cytokines such as IL-10, with the C-type lectin receptor CD206 and arginase 1 (Arg-1) as its marker [49], [50]. In the current study, we investigated the mechanisms underlying the improvement on HFD induced insulin resistance by CMHX008 with focus on adipose tissue macrophage mediated inflammation. We found that CMHX008 treatment markedly reduced serum pro-inflammatory cytokines such as TNF-α and IL-6, and elevated anti-inflammatory cytokines such as adiponectin and IL-10. To determine whether the anti-inflammatory effects of CMHX008 was associated with the change of macrophage polarization, we further examined the gene expression pattern of M1 and M2 macrophage markers. Our study showed that CMHX008 tended to decrease the expressions of CD11c, iNOS and MCP-1 and to increase the expressions of CD206 and Arg-1. These results suggest that CMHX008 may improve insulin resistance by modulating ratio of macrophage subsets, switching the polarization of adipose macrophages from pro-inflammatory M1 dominant to anti-inflammatory M2 dominant.

As several other PPARγ partial agonists under investigations, the mechanisms of differential specificity on PPARγ target genes by CMHX008 remain unclear [14], [16]. But altering the interaction of PPARγ with transcriptional coactivators is an attractive explanation. For example, PAT5A functions as a partial agonist in enhancing the interaction of PPARγ with coactivators SRC-1, CBP, PBP, and PRIP, but as a full-agonist with PGC-1α [51]. Therefore, it is likely that CMHX008 alters the ability of PPARγ to interact with its coactivators and hence promotes differential recruitment of PPARγ to the promoters of its target genes. In future studies, we will investigate mechanisms underlying the differential induction of PPARγ target genes by CMHX008.

In conclusion, CMHX008 represents a novel class of PPARγ ligand with the activity of partial agonist. CMHX008 showed a comparable effect of insulin sensitization as rosiglitazone with lower adipogenic action. Future investigations will be necessary to determine the therapeutic anti-diabetic activity, safety appraise and the molecular mechanism underlying differential regulation of PPARγ target genes by CMHX008.
